# Impact on mitral regurgitation in patients with AVB undergoing permanent pacemaker implantation: left bundle branch pacing vs. right ventricular septum pacing

**DOI:** 10.3389/fcvm.2026.1693525

**Published:** 2026-03-05

**Authors:** Liang Shan, Peng Hao, Wenlong Dai

**Affiliations:** Department of Cardiology, Beijing Anzhen Hospital, Capital Medical University, Beijing, China

**Keywords:** echocardiography, mitral regurgitation, left bundle branch pacing, left ventricular function, left ventricular remodeling, right ventricular septum pacing

## Abstract

**Background:**

Patients with atrioventricular block (AVB) who require pacemaker implantation frequently present with varying degrees of mitral regurgitation (MR), which may influence left ventricular (LV) remodeling and function. This study aimed to evaluate the impact of left bundle branch pacing (LBBP) versus right ventricular septum pacing (RVSP) on MR severity and LV performance.

**Methods:**

A total of 137 consecutive AVB patients undergoing pacemaker implantation via LBBP or RVSP were retrospectively enrolled, after excluding those with previous mitral valve surgery or structural valvular abnormalities. Baseline and follow-up echocardiography evaluations were used to assess changes in MR severity and LV function. Subgroup analyses were performed among patients with baseline MR.

**Results:**

Out of the total cohort, 88 patients (64.2%) successfully underwent LBBP, while the remaining 49 patients (35.8%) received RVSP when LBBP could not be achieved. The average age and sex distribution were similar between the two groups. Significant changes in MR area and severity at follow-up were observed in patients with LBBP compared to those with RVSP (*P* < 0.05). LBBP was independently associated with a higher likelihood of MR improvement among patients who received pacemaker implants [hazard rate (HR) = 0.238, CI = 0.105–0.538; *P* < 0.001]. At follow-up, those in the LBBP subgroup with moderate or severe MR demonstrated significantly shorter lead–TVA distance, increased left atrium diameters, poor LV ejection fraction (LVEF), and higher mitral E/A ratios (*P* < 0.05) compared with those in the none or mild MR subgroup. Among LBBP patients who showed a reduction in MR severity, there was a significant improvement in LV remodeling (ΔLVEDD) from the baseline (*P* = 0.009). Furthermore, these patients demonstrated a non-significant trend toward better LV function, reflected by greater changes in LVEF and E/A ratio (ΔLVEF and ΔE/A).

**Conclusion:**

LBBP significantly reduced MR severity and improved LV remodeling and systolic function compared with RVSP. Moreover, LBBP and baseline LVEDD were independently associated with a reduction in MR severity among AVB patients undergoing pacemaker implantation, highlighting the superiority of LBBP as a preferred pacing strategy in this population.

## Introduction

In patients with atrioventricular block (AVB), particularly those with heart failure (HF) prior to pacemaker implantation or cardiac resynchronization therapy (CRT), mitral regurgitation (MR) is a common finding. The prevalence of MR, as assessed by echocardiography, ranges widely from 8% to 74%, with HF patients showing rates between 43% and 55% (1–3). MR is strongly associated with adverse outcomes, including increased mortality and morbidity in HF patients (4). CRT—especially when delivered through physiologic pacing methods such as left bundle branch pacing (LBBP) and His-bundle pacing (HBP)—has proven to be an effective approach in improving MR among patients with wide QRS duration and left ventricular (LV) dysfunction (3, 4).

Intraventricular dyssynchrony of the myocardium is a key mechanism in causing MR (5). Several studies have validated CRT's ability to acutely and longitudinally reduce MR, although responses among HF patients vary (6, 7). HBP leads to a narrow paced QRS duration and mechanical synchrony, serving as a physiological alternative to conventional biventricular pacing within CRT. However, its efficacy is limited by high capture thresholds, lead instability, and incomplete correction of distal conduction system abnormalities (8). By contrast, LBBP is considered a near-physiological pacing method with a transventricular septal approach similar to HBP. It demonstrates excellent electrical resynchronization and a marked decrease in functional MR severity in patients with significant functional MR and left bundle branch block (LBBB) (9). Although reductions in MR severity have been observed in HBP patients with baseline grade 3 or 4 MR and left ventricular ejection fraction (LVEF) <50% using the two-dimensional biplane imaging, the study highlights potential limitations of HBP. Similarly, LBBP has shown promise in reducing MR severity, particularly in non-ischemic cardiomyopathy with LBBB (10). Nevertheless, a comprehensive comparison between LBBP and right ventricular septal pacing (RVSP) is lacking in the existing research.

Therefore, this study aims to investigate the incidence and predictive factors of MR in AVB patients with an indication for pacing therapy, and to analyze MR severity before and after pacemaker implantation, comparing the response to LBBP versus RVSP.

## Methods

### Study population

This was a prospective observational study that consecutively enrolled patients diagnosed with AVB as the sole pacing indication, with normal cardiac function, who underwent dual-chamber pacemaker implantation at Anzhen Hospital between 2018 and 2024. During the study period, LBBP was the preferred pacing strategy. Patients in whom LBBP was successfully achieved were included in the LBBP group, whereas those in whom LBBP could not be achieved underwent implantation at the right ventricular septum and were assigned to the RVSP group. Patients were excluded if they had a history of valvular heart disease or previous mitral valve surgery, atrial fibrillation, any congenital heart disease, or a history of triple-chamber pacemaker implantation. In addition, individuals below 18 years were not included in the study. Approval for the study protocol was obtained from the Ethics Committee of the Cardiovascular Institute and Anzhen Hospital (2023207X). All procedures were performed in accordance with the Declaration of Helsinki (as revised in Brazil in 2013).

### Procedural technique of LBBP

LBBP was achieved using the transventricular septal approach, in accordance with the current EHRA clinical consensus statement on conduction system pacing implantation (11). Continuous monitoring of intracardiac electrograms and 12-lead electrocardiograms was performed using an electrophysiology recording system (TOP-2001F+, APT Medical Inc.). A 3830 pacing lead (SelectSecureÔ, Model 3830, Medtronic, Minneapolis, MN, USA) was positioned on the right side of the interventricular septum (IVS) with the C315HIS sheath (Medtronic) in the right anterior oblique 30 fluoroscopic view. Unipolar (tip) pacing at 2.0 V/0.5 ms was employed to select the target site and verify optimal contact between the septum and the lead. The lead was then advanced perpendicular to the IVS and directed toward the left side of the IVS (left bundle branch area). Lead advancement, under continuous monitoring of the paced QRS morphology, persisted until predefined criteria for achieving successful LBBP were fulfilled. Confirmation of successful LBBP included paced QRS morphology showing a qR pattern in lead V1, recording of a left bundle branch potential in intracardiac electrograms, and fulfillment of other specified criteria (11, 12). Successful LBBP was defined according to the EHRA consensus statement as fulfilling all of the above-mentioned electrical and anatomical criteria. If these criteria could not be met despite multiple attempts, the pacing lead was implanted at the right ventricular septum. The patient was then assigned to the RVSP group for subsequent analysis. This strategy reflects real-world clinical practice, wherein RVSP serves as a backup when LBBP cannot be achieved.

### Measurement of MR parameters via echocardiography

Echocardiographic measurements of MR parameters were performed for all patients using an ultrasonic machine (EPIQ 7C, Philips Healthcare, Andover, MA, USA). To ensure accuracy, two experienced cardiac sonographers confirmed all echocardiographic parameters. The degree of MR was assessed both before pacemaker implantation and during follow-up. Color Doppler evaluation in standard apical four-chamber views was employed to determine the MR area. The largest regurgitant jet area during systole was manually traced and measured in cm^2^. Severity of MR was categorized into four grades (severe, moderate, mild, and none MR) according to the 2017 American Society of Echocardiography recommendations (13). Patients were then classified into two groups: group I (mild MR) included those with mild MR or no MR, and group II (significant MR) included those with moderate or severe MR. Improvement in MR was defined as a reduction of at least one grade in MR severity between baseline and follow-up assessments. During the ventricular end-diastolic phase in standard apical four-chamber views, the distance from the lead fixation site to the tricuspid annulus (Lead–TA distance) and lead depth in the IVS were measured in patients with LBBP.

### Data collection and follow-up

Prospective evaluation of clinical status, LV function, and MR was performed at baseline (within 1 week prior to pacemaker implantation) and during subsequent follow-ups. Collected data included left atrial (LA) diameters, IVS thickness and motion amplitude, LV end-diastolic and end-systolic diameters, LVEF using the two-dimensional biplane-modified Simpson approach, and mitral E/A ratio. Follow-ups were performed at 1 month, 3 months, and every 6 months thereafter in the device clinic via echocardiography. The endpoint was change in MR severity.

### Time-to-event definition

The time-to-event variable was defined as the interval (in days) from pacemaker implantation to the first documented improvement in MR severity (a decrease of ≥1 grade on echocardiography). Patients without MR improvement during follow-up were censored at the time of their last echocardiographic evaluation.

### Statistical analyses

Data analyses were performed using SPSS v25.0 (IBM Inc., New York). Continuous variables were presented as mean ± SD and compared using the Student’s *t*-test. Categorical variables, expressed as percentage, were analyzed using the chi-squared test. The Kaplan–Meier method assessed the association of changes in MR, with comparisons made using the log-rank test. Cox proportional hazards regression models were employed to determine independent factors associated with changes in MR. Statistical significance was defined as *P* < 0.05.

## Results

### Baseline clinical features

During the study period, 137 patients diagnosed with AVB who received pacemakers delivered by LBBP or RVSP were enrolled in the cohort. Among these, 88 patients (mean age 68 ± 13 years; 62.5% male) underwent successful LBBP. In the remaining 49 patients (mean age 72 ± 11 years; 61.2% male), LBBP could not be achieved due to anatomical or technical limitations, and RVSP was performed instead. This reflects a procedural success/failure model rather than a preassigned group allocation. Baseline demographics and clinical features of the entire cohort are presented in Table 1. Notably, there were no obvious differences in baseline clinical characteristics between the two pacing groups. The median follow-up period for the cohort was 146 (94, 339) days, with the average follow-up duration being comparable between the two groups (*P* = 0.112).

**Table 1:** Baseline features of the total samples.

Variable	LBBAP (*n* = 88)	RVSP (*n* = 49)	*P*-value
Age (years)	68 ± 13	72 ± 11	0.107
Male (%)	55 (62.5)	30 (61.2)	0.883
BNP	159.50 (92.25, 249.75)	160.50 (82.50, 324.00)	0.846
Comorbidity			
CAD (%)	41 (46.6)	18 (36.7)	0.264
HT (%)	54 (61.4)	31 (63.3)	0.826
DM (%)	30 (34.1)	12 (24.5)	0.243
HP (%)	46 (52.3)	23 (46.9)	0.550
Stroke (%)	8 (9.1)	6 (12.2)	0.559
Pacing Indications			
AVB (%)	88 (100.0%)	49 (100.0%)	-
Medication			
ACEI/ARB (%)	40 (45.5)	20 (40.8)	0.561
*β* (%)	25 (28.4)	11 (22.4)	0.447
CCB (%)	30 (34.1)	22 (44.9)	0.230
Echocardiographic Data			
LA (mm)	41.5 ± 6.6	42.4 ± 6.2	0.447
IVS (mm)	10.2 ± 2.1	9.9 ± 3.1	0.602
IVS motion amplitude	7.2 ± 2.0	7.3 ± 1.5	0.704
LVEDD (mm)	49.1 ± 6.9	47.6 ± 4.9	0.128
LVESD (mm)	32.6 ± 6.9	31.1 ± 4.8	0.175
LVEF (%)	60.9 ± 8.5	63.2 ± 6.9	0.105
E/A	1.4 ± 1.6	1.2 ± 0.8	0.404

ACEI, angiotensin-converting enzyme inhibitor; ARB, angiotensin II receptor blocker; CAD, coronary heart disease; CCB, calcium channel blocker; DM, diabetes mellitus; HP, hyperlipidemia; HT, hypertension.

### Echocardiographic comparison between LBBP and RVSP

**Figure 1:**
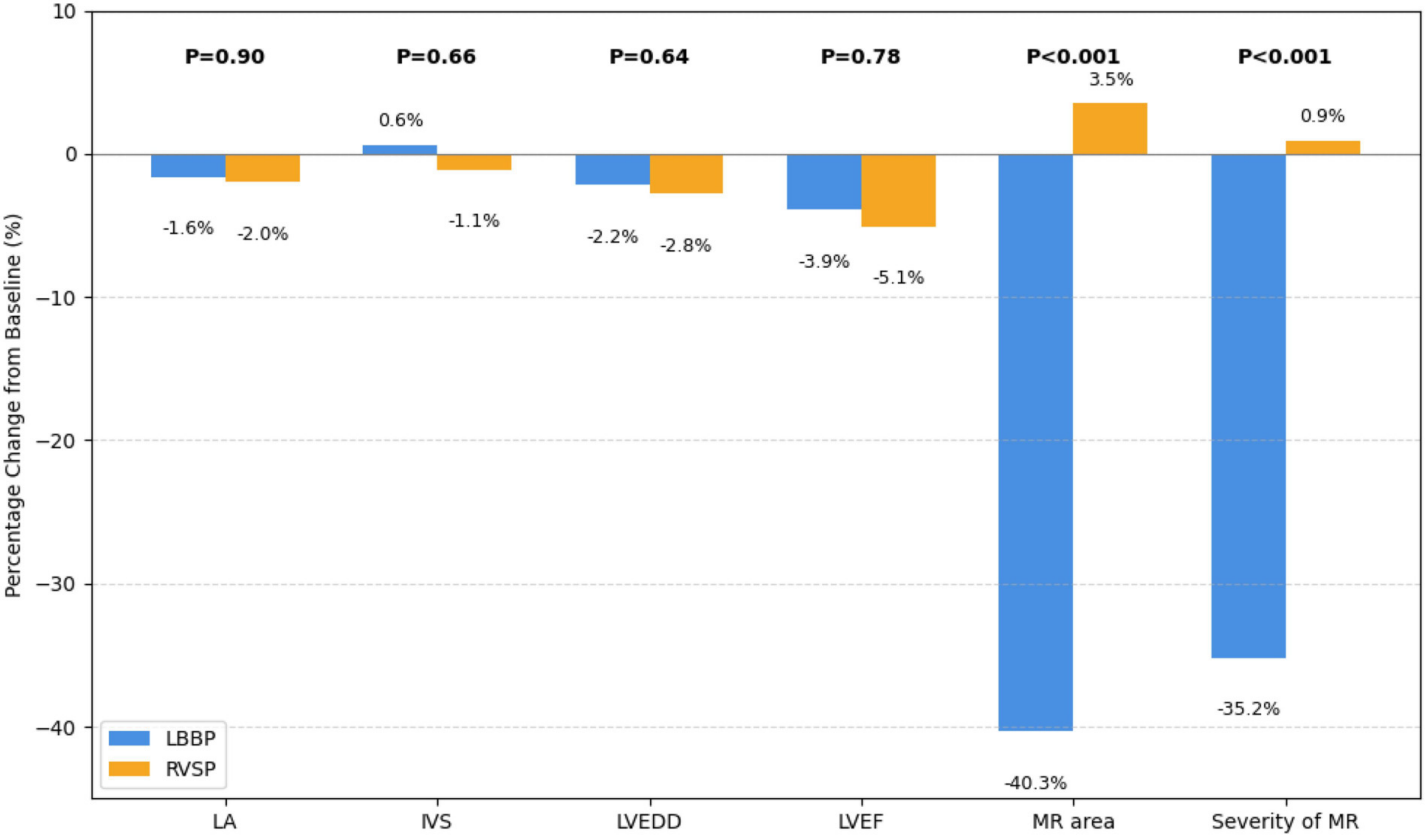
Percentage changes in echocardiographic parameters between the LBBP and RVSP groups.

The comparison of echocardiographic results at baseline and follow-up is presented in Table 2. Notably, there were no obvious differences in baseline echocardiographic features between the LBBP and RVSP groups. However, at the follow-up assessment, LBBP exhibited a notable improvement in MR compared with RVSP. Specifically, 16 out of 88 patients (18.2%) treated with LBBP showed significant MR, compared with 23 out of 49 patients (46.9%) in the RVSP group (*P* < 0.001). The MR area decreased from 4.5 ± 4.3 to 2.7 ± 3.3 cm^2^ in the LBBP group, with a significant difference compared to RVSP at the last follow-up (*P* = 0.013). When assessing the magnitude of change, LBBP showed a distinct impact on the MR area (LBBP: 0.4 ± 1.6 cm^2^ vs. RVSP −0.3 ± 2.3 cm^2^; *P* < 0.001) and a similar improvement in MR severity (LBBP: 0.5 ± 0.7 vs. RVSP −0.1 ± 0.8; *P* < 0.001). There was a non-significant trend indicating greater enhancement in LA diameters, left ventricular end-diastolic diameters (LVEDD), LVEF, and IVS motion amplitude in the LBBP group. As shown in Figure 1, LBBP led to a significant percentage reduction in MR area and severity of MR. Both groups exhibited slight decreases in LA diameters, LVEDD, LVEF, and IVS motion amplitude, with no significant differences between them.

**Table 2:** Comparison of echocardiographic results at baseline and follow-up between the LBBP and RVSP groups.

Variable	LBBP (*n* = 88)	RVSP (*n* = 49)	*P*-value
Baseline			
LA (mm)	68 ± 13	68 ± 13	0.447
IVS (mm)	72 ± 11	72 ± 11	0.602
IVS motion amplitude	41.5 ± 6.6	41.5 ± 6.6	0.704
LVEDD (mm)	42.4 ± 6.2	42.4 ± 6.2	0.128
LVESD (mm)	10.2 ± 2.1	10.2 ± 2.1	0.175
LVEF (%)	9.9 ± 3.1	9.9 ± 3.1	0.105
E/A	7.2 ± 2.0	7.2 ± 2.0	0.404
MR area (cm^2^)	4.5 ± 4.3	3.7 ± 3.20	0.278
Significant MR	38	21	0.971
Follow-up			
LA (mm)	40.8 ± 7.1	41.5 ± 5.4*	0.552
IVS (mm)	10.5 ± 2.4*	10.8 ± 3.3*	0.518
IVS motion amplitude	6.9 ± 1.6*	6.9 ± 1.7*	0.968
LVEDD (mm)	48.1 ± 6.8*	46.5 ± 4.5*	0.102
LVESD (mm)	32.6 ± 7.5*	30.9 ± 5.2*	0.174
LVEF (%)	58.5 ± 9.2*	59.9 ± 9.0*	0.400
E/A	1.1 ± 0.6	0.9 ± 0.4*	0.245
MR area (cm^2^)	2.7 ± 3.3*	4.1 ± 2.7	0.013
Significant MR	16	23	<0.001
Changed values, *Δ* (pre–post)			
*Δ*LA (mm)	0.7 ± 4.3	0.9 ± 5.0	0.798
*Δ*IVS (mm)	−0.3 ± 1.9	−0.8 ± 1.6	0.114
*Δ*IVS motion amplitude	0.4 ± 2.3	0.3 ± 1.9	0.656
*Δ*LVEDD (mm)	1.1 ± 6.5	1.1 ± 4.2	0.996
*Δ*LVESD (mm)	0.1 ± 6.4	0.4 ± 4.1	0.787
*Δ*LVEF (%)	2.2 ± 7.4	2.4 ± 7.7	0.895
*Δ*E/A	0.2 ± 0.8	0.3 ± 0.7	0.717
*Δ*MR area (cm^2^)	0.4 ± 1.6	−0.3 ± 2.3	<0.001
*Δ*Severity of MR	0.5 ± 0.7	−0.1 ± 0.8	<0.001

* *P*-values derived using paired *t*-test.

### Potential factors associated with changes in MR in patients who underwent pacemaker implantation

**Figure 2:**
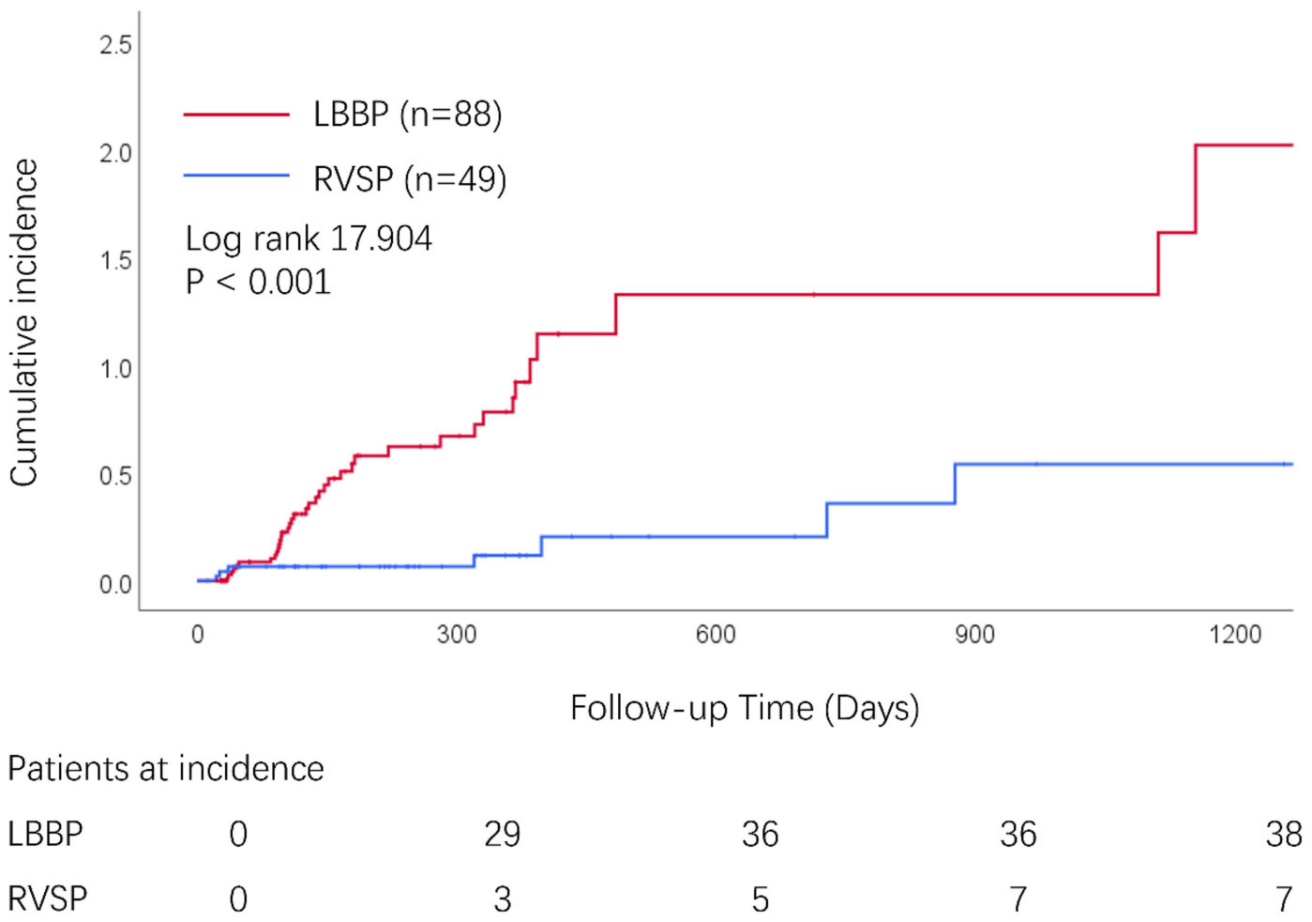
Kaplan–Meier curves for the values and incidence of MR reduction after LBBP or RVSP treatment strategy.

The impact of LBBP on the cumulative incidence of MR severity reduction was assessed following device implantation (*P* < 0.001; Figure 2). The variables that were significantly associated with changes in MR are displayed in Table 3. Univariate Cox regression analysis showed that both the implantation procedure and LVEDD were significantly associated with MR change after pacemaker implantation. In the multivariate Cox model, the LBBP pacing strategy [hazard rate (HR) = 0.238, 95% CI: 0.105–0.538; *P* < 0.001] and larger baseline LVEDD (HR = 1.054, 95% CI: 1.009–1.101; *P* = 0.018) remained independently associated with the time to MR improvement in AVB patients who underwent pacemaker implantation.

**Table 3:** Univariate and multivariable Cox regression models for changes in MR in patients who underwent pacemaker implantation.

Variable	Univariate model		Multivariate model	
	HR (CI)	P	HR (CI)	P
Age	1.014 (0.990–1.040)	0.248		
Male	0.847 (0.455–1.575)	0.599		
BNP	1.000 (0.999–1.001)	0.813		
CAD	1.432 (0.794–2.581)	0.233		
HT	1.329 (0.706–2.502)	0.378		
HP	1.200 (0.665–2.166)	0.545		
Implantation procedure	0.205 (0.091–0.460)	<0.001	0.238 (0.105–0.538)	<0.001
LA	1.026 (0.982–1.073)	0.255		
IVS	1.023 (0.918–1.141)	0.678		
IVS motion amplitude	0.959 (0.801–1.148)	0.646		
LVEDD	1.070 (1.024–1.118)	0.003	1.054 (1.009–1.101)	0.018
LVEF	0.969 (0.933–1.006)	0.101		
E/A	0.819 (0.535–1.254)	0.359		

### Effects of MR on LV remodeling and function in LBBP subgroup at follow-up

A total of 88 patients (64.2%) underwent LBBP implantation for AVB. At the last follow-up, the LBBP cohort was categorized into two subgroups: group I (*n* = 72) with mild MR or no MR and group II (*n* = 16) with moderate or severe MR. Compared with mild MR patients, significant MR patients had significantly shorter lead–TVA distance, lower LVEF, and higher LA diameters and mitral E/A ratio (*P* < 0.05). No differences were observed in lead depth and other electrocardiography indices between groups I and II (Table 4).

**Table 4:** Comparison between mild MR and significant MR in LBBP subgroup at follow-up.

Variable	Mild MR (*n* = 72)	Significant MR (*n* = 16)	*P*-value
Lead position			
Lead–TVA distance (mm)	26.6 ± 10.7	15.1 ± 6.3	0.034
Lead depth (mm)	11.3 ± 3.4	8.8 ± 2.5	0.099
Follow-up			
LA (mm)	39.7 ± 6.9	45.7 ± 6.2	0.003
IVS (mm)	10.5 ± 2.6	10.5 ± 1.8	0.985
IVS motion amplitude	6.9 ± 1.6	6.6 ± 1.3	0.489
LVEDD (mm)	47.7 ± 6.9	49.9 ± 6.3	0.252
LVESD (mm)	31.9 ± 7.5	35.7 ± 7.1	0.231
LVEF (%)	59.9 ± 8.6	52.4 ± 9.7	0.003
E/A	0.9 ± 0.5	1.8 ± 0.8	<0.001

**Figure 3:**
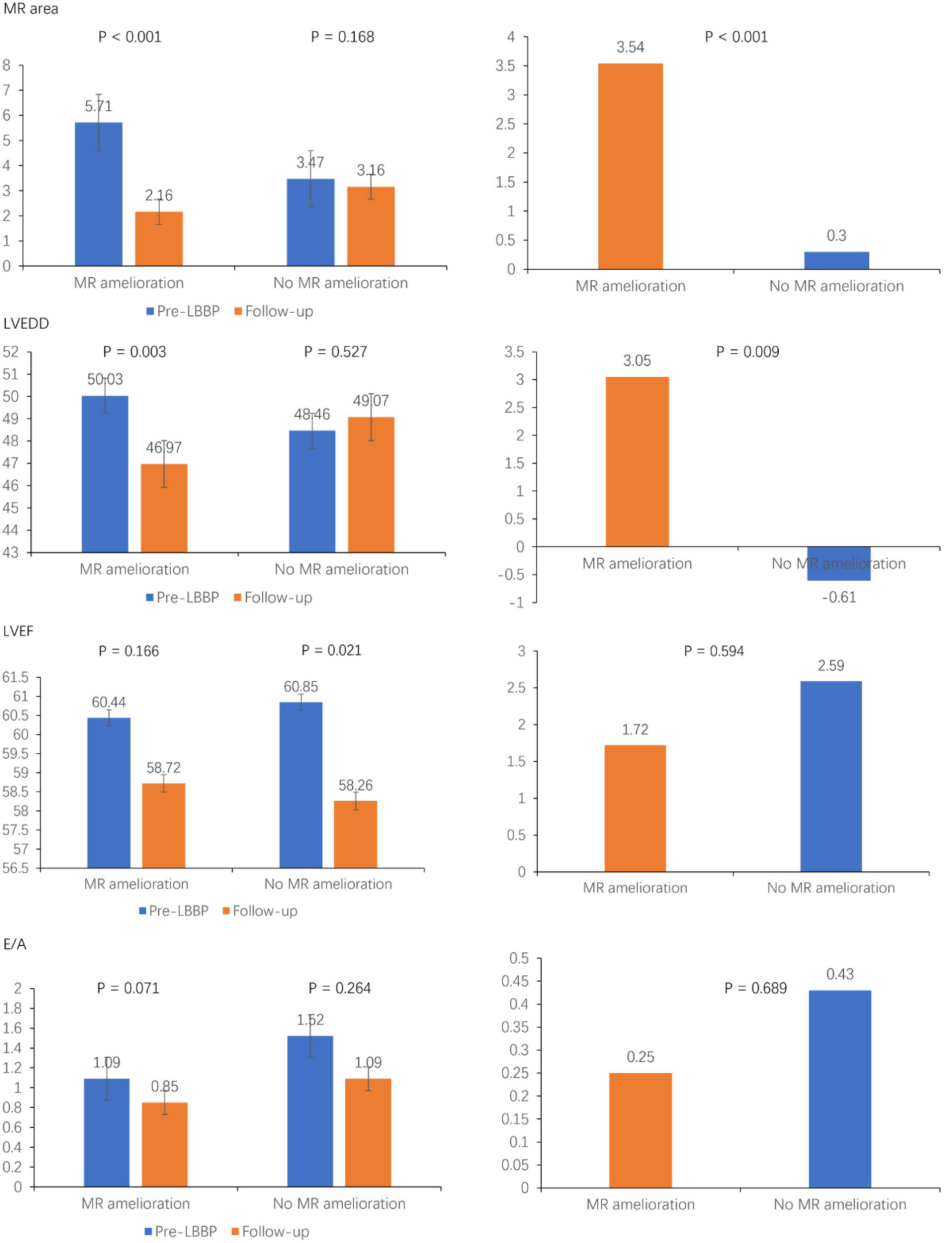
Severity of echocardiographic changes in LBBP patients stratified by baseline MR severity.

During follow-up, 41 patients (46.6%) in the LBBP group exhibited a reduction in MR severity. LBBP resulted in a reduction in MR area from 5.7 ± 3.2 cm^2^ to 2.16 ± 1.99 cm^2^ (*P* < 0.001), and LVEDD decreased from 50.0 ± 6.7 mm to 47.0 ± 5.2 mm (*P* = 0.003) in MR reduction. In patients with MR without amelioration, LVEF decreased from 60.8 ± 8.8 mm to 58.3 ± 10.0 mm (*P* = 0.021). On the contrary, LV remodeling (*Δ*LVEDD) was significantly improved in the MR reduction group (*P* = 0.009). Furthermore, there was a non-significant trend toward greater enhancement of LV function (ΔLVEF and ΔE/A) in patients with MR reduction (Figure 3).

### Incidence of atrial fibrillation during follow-up

During the follow-up period, four patients (4.5%) in the LBBP group and five patients (10.2%) in the RVSP group developed new-onset atrial fibrillation (AF) (Table 5). Although the incidence was numerically higher in the RVSP group, the difference did not reach statistical significance (*P* = 0.200). These events were identified through routine electrocardiogram follow-up and pacemaker interrogation records.

**Table 5:** Clinical events during follow-up.

Variable	LBBP (*n* = 88)	RVSP (*n* = 49)	*P*-value
New-onset AF (%)	4 (4.5%)	5 (10.2%)	0.200

## Discussion

This study systematically assessed the impact of LBBP and RVSP on echocardiographic measures of MR. The primary findings of our investigation can be summarized as follows: (1) Compared with RVSP, LBBP was associated with a greater reduction in MR area and severity from baseline to follow-up, whereas no significant difference was observed at baseline between the two groups. (2) LBBP was associated with changes in MR among patients undergoing pacemaker implantation. (3) Within the LBBP subgroup, patients with moderate or severe MR at follow-up exhibited a significantly shorter lead–TVA distance. (4) In patients with LBBP and MR reduction, there was a discernible trend toward enhanced LV remodeling and function.

MR, often secondary to LV dysfunction, is a prevalent concern, estimated to affect 43%–55% of patients (14). In our study, 55% of patients with AVB presented with moderate or severe MR. The association between MR, deteriorated LV function, and adverse prognosis is well established, particularly in HF patients (4). Increasing research has identified the link between severe MR and major adverse events (15). In a previous study of 1,421 patients with LV dysfunction, 18.9% had severe MR, with an additional 84% increased risk of death (16). Therefore, MR is not merely a consequence of LV remodeling but also a crucial indicator of poor clinical outcomes. The distinction between mild and severe MR is crucial, as severe MR can significantly impact stroke volume, especially in the presence of impaired systolic function (17). Currently, there are many remaining challenges ahead in the field of MR management. Treatment of MR is primarily directed toward its underlying cause, which is a dysfunction of the left ventricle. Optimizing the use of resynchronization devices is pivotal, as they have the potential to reverse adverse remodeling processes, subsequently reducing LV volumes and ameliorating MR severity (18, 19).

The primary cause of MR is attributed to discoordination among the myocardial segments proximal to the papillary muscles (5). Extensive research has established the effectiveness of biventricular pacing (BVP) in acutely reducing MR and maintaining these benefits during follow-up (6, 7). Prior research has demonstrated the improvement in MR severity with BVP, with a distinct correlation between MR response and favorable outcomes in CRT (3). Narrow paced QRS duration and myocardial synchrony make HBP a compelling alternative to BVP. Research has demonstrated that HBP not only reduces MR but also results in a narrower paced QRS duration, particularly in MR responders (20). A novel form of physiological pacing, LBBP addresses limitations associated with HBP, including high capture thresholds and suboptimal correction of the distal conduction system. Huang et al. demonstrated a remarkable decrease in QRS duration and enhancement in LVEF with LBBP (21). LBBP has been shown to significantly reduce functional MR severity without worsening MR from baseline. Moreover, LBBP led to similar decreases in QRS duration, LVEDD, and LVEF (10). In our study, compared to RVSP, LBBP exhibited significant improvements and a consistent percentage reduction in both MR area and MR severity from baseline to follow-up. Furthermore, LBBP was associated with changes in MR in patients undergoing pacemaker implantation.

Although the exact pathophysiological mechanisms of LBBP benefits remain unclear, they are likely multifaceted, impacting the degree of MR, LV remodeling, and potentially LV function. Notably, LBBP was associated with higher LA diameters and mitral E/A ratio in patients with moderate or severe MR. However, in cases where MR was ameliorated with LBBP, LV remodeling and function showed improvement at follow-up, consistent with prior research (10). Assessing the impact of pacing on LV systolic and diastolic function poses challenges, given the intricate nature of these echocardiographic parameters. Their assessment is influenced by diverse factors, including cardiac rhythm and MR. Notably, in our study, physiological pacing exhibited a more prominent effect, marked by a reduction in MR severity, consistent with decreases in pulmonary pressure observed in acute studies (22). This observation highlights that RVSP has the potential to induce and exacerbate MR, whereas the delayed effect witnessed is attributed to reverse LV remodeling, contributing to a further reduction in MR severity (4). The significant reduction in MR area observed in our study underscores the favorable impact of LBBP on the intricate relationships between the mitral valve, LV remodeling, and LV function in patients with AVB. Thus, optimal utilization of LBBP holds the potential to reverse the LV remodeling process, leading to a subsequent reduction in LV volumes and further amelioration of MR severity. Historically, the management of secondary or functional MR has primarily focused on restoring LV structure and function through drugs or devices, avoiding direct surgical correction (17). Pending further validation through additional studies, our findings position LBBP as a promising therapeutic option for patients with severe MR undergoing pacemaker implantation (23).

### Limitations

Despite the valuable insights gained, our research has several limitations. This was a single-center observational study, inherently subject to observational and selection biases. However, each subgroup assignment for LBBP or RVSP was closely matched in terms of clinical baseline characteristics. Patients were not randomly assigned to a pacing strategy; rather, the comparison reflects a procedural success model instead of a randomized allocation. LBBP was attempted first, and in cases where LBBP could not be successfully achieved, the pacing lead was implanted at the right ventricular septum and the patient was assigned to the RVSP group. This approach means that patients in whom conduction system pacing could not be achieved may have had underlying anatomical or conduction characteristics that both increased the likelihood of LBBP failure and were independently associated with more severe mitral regurgitation. Therefore, part of the observed differences between groups may reflect underlying substrate rather than the pacing modality alone. However, this reflects real-world clinical practice and provides valuable practical insights into the comparative outcomes of the two pacing modalities. In addition, the relatively small sample size was another limitation, although it represents the primary implantation procedure in patients with AVB. Finally, the mechanisms underlying the benefits of LBBP on MR, LV remodeling, and LV function remain unclear and necessitate confirmation through further research. The promising results highlighted in this open pilot study require validation through larger-scale studies, particularly in terms of other causes of MR beyond AVB.

## Conclusion

In this real-world procedural cohort, patients who underwent successful conduction system pacing, specifically LBBP, demonstrated a significantly greater reduction in MR severity and improved LV remodeling compared with those in whom conduction system pacing could not be achieved and who subsequently received RVSP. Successful LBBP implantation and larger baseline LVEDD were independently associated with MR improvement during follow-up. These findings suggest that LBBP may serve as a favorable pacing strategy for patients with atrioventricular block, although the observed benefits may partly reflect patient characteristics associated with successful implantation.

## Data Availability

The original contributions presented in the study are included in the article/Supplementary Material, further inquiries can be directed to the corresponding author.
